# *Mucuna pruriens* Treatment for Parkinson Disease: A Systematic Review of Clinical Trials

**DOI:** 10.1155/padi/1319419

**Published:** 2025-08-18

**Authors:** Fatima Hammoud, Ali Ismail, Reem Zaher, Rania El Majzoub, Linda Abou-Abbas

**Affiliations:** ^1^Faculty of Medical Sciences, Neuroscience Research Center, Lebanese University, Beirut, Lebanon; ^2^Faculty of Medical Sciences, Lebanese University, Beirut, Lebanon; ^3^School of Pharmacy, Department of Biomedical Sciences, Lebanese International University, Mazraa 146404, Lebanon; ^4^INSPECT-LB (Institut National de Santé Publique Epidémiologie Clinique et Toxicologie-Liban), Beirut, Lebanon

**Keywords:** L-dopa, levodopa, *M. pruriens*, *Mucuna pruriens*, Parkinson disease, systematic review

## Abstract

**Background:** Research into alternative treatments for Parkinson's disease (PD) is gaining increasing attention. *Mucuna pruriens* (*M. pruriens*), a plant traditionally used in Ayurvedic medicine, contains a significant amount of L-dopa (4%–6%), the primary active component of conventional levodopa (LD) therapy—the gold standard treatment for PD. *M. pruriens* is also recognized for its anti-inflammatory, antioxidant, antiapoptotic, and antiparkinsonian properties, which collectively suggest therapeutic benefits for individuals with PD.

**Objective:** This systematic review aims to investigate the efficacy and safety of *M. pruriens* in managing symptoms of PD.

**Methods:** A comprehensive search was conducted in PubMed, Embase, and Web of Science for clinical trials published up to February 2024. Studies comparing *M. pruriens* to LD were included. Quality assessment was performed, and findings were synthesized narratively.

**Results:** Out of 466 articles identified, 5 clinical trials involving a total of 108 participants (mean age: 60 years) were included. Quality assessment rated one study as high quality, one as having some concerns, and three as low quality. Despite heterogeneity in *M. pruriens* interventions, the findings consistently showed improvements in PD symptoms and therapy-related complications. Treatment with *M. pruriens* was associated with a shorter time to reach the “on” disease stage, prolonged duration of this stage, and fewer adverse events, with no dyskinesia reported.

**Conclusion:** *M. pruriens* shows promise in improving motor symptoms and reducing therapy complications in PD patients. However, current clinical evidence is limited, and further high-quality trials are needed to confirm its efficacy and safety.

## 1. Introduction

Parkinson's disease (PD) is a neurodegenerative disorder characterized by the progressive degeneration of dopaminergic neurons in the substantia nigra pars compacta. This degeneration disrupts the nigrostriatal pathway, a critical neural circuit responsible for regulating voluntary movement, leading to dopamine dysregulation and impaired motor control [1–4]. Clinically, PD manifests as resting tremor, bradykinesia, muscular rigidity, and postural instability, alongside nonmotor symptoms such as depression, sleep disturbances, and cognitive decline [5, 6].

At the cellular level, PD is characterized by two key pathological processes: The loss of dopaminergic neurons and the accumulation of misfolded *α*-synuclein protein aggregates, which form Lewy bodies. These aggregates disrupt neuronal homeostasis and contribute to neuroinflammation, mitochondrial dysfunction, and oxidative stress [2, 7]. Under normal conditions, *α*-synuclein resides in the cytoplasm of neurons and glial cells; however, its pathological aggregation plays a central role in the neurodegenerative processes observed in PD [8]. Oxidative stress, resulting from an imbalance between reactive oxygen species (ROS) and the brain's antioxidant defenses, triggers lipid peroxidation, protein misfolding, and neuronal apoptosis, making it a major pathogenic mechanism and promising therapeutic target in PD and other neurodegenerative diseases [8].

Levodopa (LD), as a dopamine precursor, remains the cornerstone of PD pharmacotherapy because it replenishes the deficient dopamine caused by neuronal loss [9, 10]. When administered orally, LD crosses the blood–brain barrier and is converted into dopamine in the brain, improving motor symptoms such as tremor, rigidity, and bradykinesia. However, LD is rapidly metabolized in peripheral tissues before reaching the brain, necessitating its coadministration with dopa-decarboxylase inhibitors (DDCIs) or catechol-O-methyltransferase (COMT) inhibitors to increase bioavailability and reduce peripheral side effects [11]. Despite these advances that enhance symptomatic relief, LD does not halt disease progression [11].

Moreover, long-term LD therapy is associated with motor complications and reduced efficacy [10, 12]. A hallmark of these complications is the fluctuation between “on” and “off” phases: The “on phase” refers to periods when LD effectively manages symptoms and motor function improves, while the “off phase” denotes times when the medication's effects wane, causing the re-emergence of symptoms such as tremor, rigidity, or bradykinesia [12, 13]. These fluctuations, along with systemic side effects, continue to impact patients' quality of life (QoL), underscoring the need for alternative and complementary therapeutic strategies.

In response to these limitations, research has expanded toward alternative and complementary treatment modalities that may provide more sustained symptom control or neuroprotective effects. Among experimental strategies, neuromodulation techniques such as spinal cord stimulation (SCS) have shown promise in alleviating motor impairments in preclinical PD models. Studies by Yadav et al. and Santana et al. showed that SCS not only improves motor deficits but may also preserve dopaminergic function in animal models [14, 15]. These encouraging findings reflect a broader effort to explore nonpharmacological and biologically diverse therapies that extend beyond dopamine replacement.

Within this context, plant-based interventions have gained attention for their potential therapeutic value. *Mucuna pruriens* (L.), in particular, stands out due to its high natural L-dopa content and a range of pharmacologically active compounds, offering a promising complementary strategy for the management of PD symptoms [16, 17]. Belonging to the Fabaceae family and Papilionaceae subfamily, *M. pruriens* is native to tropical and subtropical regions such as southern China and India [18, 19]. All parts of the plant are reported to possess medicinal value; however, the seeds are particularly rich in natural L-dopa (4%–6%) along with other bioactive compounds such as serotonin, NADH, phenolics, and coenzyme Q10 [17, 20–22]. The leaves also contain alkaloids, phenolic acids, and triterpenes like ursolic acid and betulinic acid. These components are associated with a broad range of pharmacological effects, including antioxidant, anti-inflammatory, neuroprotective, and antiapoptotic activities [17, 21, 23, 24].

A growing body of preclinical research has confirmed the efficacy of *M. pruriens* in PD models. Studies have demonstrated that its extracts exert antioxidant effects, preserve mitochondrial function, and improve motor behavior. For instance, acidic extracts rich in phenolics and L-dopa have shown significant neuroprotective properties, while cotyledon extracts appear to restore neurotransmitter balance in the nigrostriatal pathway [25]. Additionally, *M. pruriens* has been found to enhance mitochondrial complex I activity and reduce oxidative stress through the actions of its phytoestrogens and cofactors. Importantly, its use has not been associated with the long-term complications commonly observed with synthetic LD.

A systematic review by Francisca Idalina Neta emphasized the translational relevance of *M. pruriens*, compiling evidence from experimental PD models and confirming its therapeutic promise in modulating oxidative stress and dopaminergic function [25]. Including such preclinical data strengthens the rationale for exploring *M. pruriens* as a complementary treatment in human PD populations [25].

Given the pharmacological potential of *M. pruriens* and the need for safer, more sustainable PD therapies, this systematic review aims to evaluate the efficacy and safety of *M. pruriens* in the management of PD symptoms, focusing on its motor, neuroprotective, and oxidative stress-related effects.

## 2. Methods

### 2.1. Protocol and Registration

The systematic review proposal was registered with the International Prospective Register of Systematic Reviews under the registration number CRD42024510951. This review was conducted in accordance with the Preferred Reporting Items for Systematic Reviews and Meta-Analyses 2020 (PRISMA 2020) guidelines [26].

### 2.2. Eligibility Criteria

Studies were selected based on predefined inclusion and exclusion criteria.

#### 2.2.1. Inclusion Criteria

1. Clinical trials involving patients diagnosed with PD who were treated with either LD or *M. pruriens.*
2. Both randomized controlled trials (RCTs) and nonrandomized interventional clinical trials were included to capture a broad range of clinical evidence.
3. Studies involving human participants.
4. Publications written in English with full-text access available.

#### 2.2.2. Exclusion Criteria

1. Studies conducted on cell cultures or animal models.
2. Studies that assessed pharmacological interventions other than LD or *M. pruriens.*
3. Nonpeer-reviewed sources, including books, newspaper articles, and reports.

### 2.3. Search Strategy

A comprehensive systematic search was conducted in the following electronic databases: PubMed, Embase, and Web of Science, covering all records from each database's inception through February 2024.

Two independent reviewers performed the searches using combinations of Medical Subject Headings (MeSH) terms and keywords. The search strategy focused on two main domains:

• *M. pruriens*-related terms: Mucunas; Bean, Velvet; Beans, Velvet; Velvet Bean; Velvet Beans; Cowitch; Cowitches
• PD-related terms: Idiopathic Parkinson's Disease; Lewy Body Parkinson's Disease; Parkinson's Disease, Idiopathic; Parkinson's Disease, Lewy Body; Parkinson Disease, Idiopathic; Parkinson's Disease; Idiopathic Parkinson Disease; Lewy Body Parkinson Disease; Primary Parkinsonism; Parkinsonism, Primary; Paralysis Agitans

Boolean operators (“OR” within each category and “AND” between the two categories) were used to ensure a sensitive and comprehensive search.

### 2.4. Selection Process

All retrieved citations were imported into Zotero reference management software to identify and remove duplicates. The screening process was then carried out using Rayyan software [27]. Two independent reviewers screened the titles and abstracts of the studies based on the eligibility criteria. Full-text articles were retrieved for all records deemed potentially relevant. The same reviewers independently assessed the full texts for final inclusion. Any disagreements or discrepancies were resolved through discussion and consensus with a third reviewer when necessary.

### 2.5. Data Collection Process

Data extraction was conducted independently by two reviewers using a predesigned data extraction form in Microsoft Excel. The extracted information included the study title and authors, year of publication, number and age range of participants, study design, disease duration, type of intervention (LD or *M. pruriens*), reported outcomes related to efficacy and/or safety, and the main findings concerning the management of PD symptoms.

### 2.6. Risk of Bias Assessment

The risk of bias was assessed according to the design of the included studies. Studies were categorized based on their design into RCTs and nonrandomized clinical trials. RCTs are defined as studies in which participants were randomly allocated to intervention groups, reducing selection bias and balancing confounders. Nonrandomized clinical trials included interventional studies without random allocation, where assignment to treatments was based on other criteria.

Accordingly, the Revised Cochrane Risk of Bias Tool (RoB 2) was applied to assess the quality of RCTs, evaluating five domains: bias arising from the randomization process, deviations from intended interventions, missing outcome data, outcome measurement, and selection of reported results [28]. For nonrandomized clinical trials, the Risk of Bias in Non-randomized Studies of Interventions (ROBINS-I) tool was used, assessing seven bias domains including confounding, participant selection, intervention classification, deviations from intended interventions, missing data, outcome measurement, and selection of reported results [29].

### 2.7. Data Synthesis

Due to the heterogeneity in study designs, intervention protocols, and outcome measures across the included trials, a narrative synthesis was conducted. Study characteristics and key findings were summarized in tabular format to enable cross-study comparisons. Risk of bias findings were presented as visual diagrams.

## 3. Results

### 3.1. Study Selection

**Figure 1:**
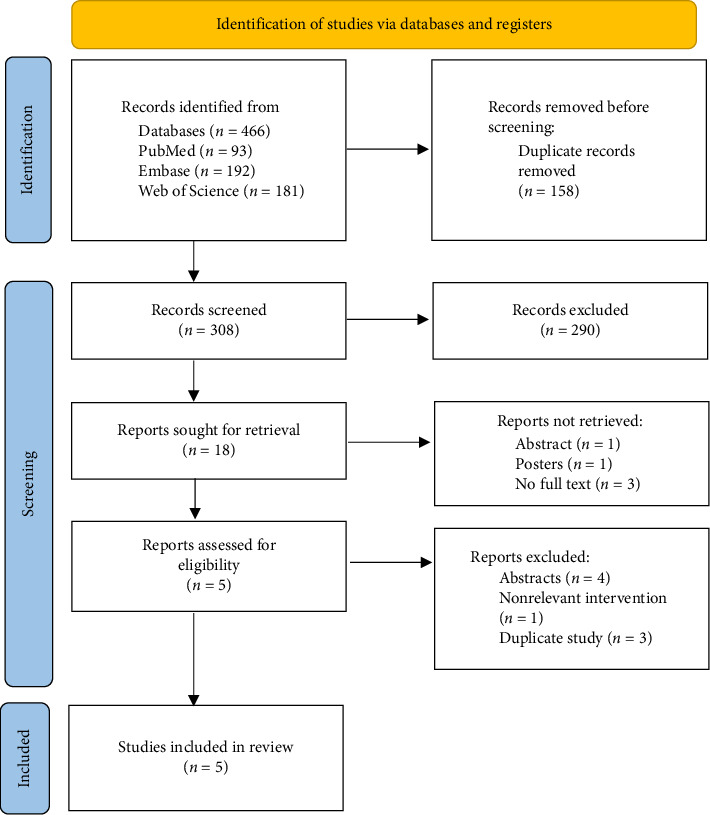
PRISMA flowchart of included articles in our systematic review.

A total of 466 records were initially identified through database searches. After removing 158 duplicates using Zotero reference management software, 308 articles remained for screening. These were imported into Rayyan software for title and abstract screening, which led to the exclusion of 290 noneligible articles. The full texts of the remaining 18 articles were then assessed for eligibility. Of these, 5 studies were included in the final analysis. The remaining 13 articles were excluded for the following reasons: 5 articles could not be retrieved, 3 had an incorrect study design, 4 involved nonrelevant interventions, and 1 was a duplicate. Please refer to the PRISMA flow diagram (Figure 1) for further details.

### 3.2. Study Characteristics

The characteristics of included studies are shown in Table 1. Five trials published between 1995 and 2024 were selected for inclusion, involving 108 participants allocated to the experimental group, with sample sizes ranging from 7 to 60 patients. The mean age was 60 years, with disease duration varying between 4.2 and 12.4 years. Regarding treatment used, patients received LD alone [31, 33] or in combination with carbidopa (CD), dopamine agonists, monoamine oxidase-B inhibitors (iMAO-B), catechol-O-methyltransferase inhibitors (iCOMTs), amantadine, or anticholinergics [32]. They then received *M. pruriens* plant preparations, with varying doses across studies. In the trial conducted in 1995, HP-200 extract from *M. pruriens*, which contains 33.33 mg of LD per gram, was administered as sachets of 1.5 g each, with intake ranging from 3 to 7 sachets [30]. In the Katzenschlager et al. trial in 2004, *M. pruriens* was given at doses of 15 g (≈500 mg LD) or 30 g (≈1000 mg LD) compared to 200 mg levodopa/50 mg carbidopa (standard LD/CD) [31]. In the trial conducted by Cilia et al. in 2017, two doses of *M. pruriens* were administered: a low dose containing 12.5 mg/kg of LD and a high dose containing 17.5 mg/kg of LD [32], and in their trial in 2018, *M. pruriens* was administered with a 4144 mg/day dose of LD consistent with the 5.7% calculation. The Sakata et al. trial administered 11 g of *M. pruriens containing* 442.2 mg of LD [34]. Despite the heterogeneity in treatments, studies evaluated the efficacy of *M. pruriens* using clinical assessments, including the area under the plasma concentration curve “AUC,” the peak plasma LD concentration “*C*_max_,” the time to peak “*T*_max_” and the apparent elimination half-time “*T*_*k*_.” Various scales were also used to assess changes in disease symptoms. For motor response, the Unified Parkinson's Disease Rating Scale (UPDRS), the Abnormal Involuntary Movement Scale (AIMS), and the Goetz rating scale were used; for nonmotor response, the Non-Motor Symptoms Questionnaire (NMSQ) was applied. QoL was measured with the Parkinson's Disease Questionnaire-39 (PDQ-39), and disability was evaluated using the Hoehn and Yahr scale (H&Y). Safety outcomes were assessed by monitoring adverse events (AEs) following *M. pruriens* treatment.

Table 2 displays Parkinson's disease symptoms studied in each trial.

### 3.3. Risk of Bias in Studies

**Figure 2:**
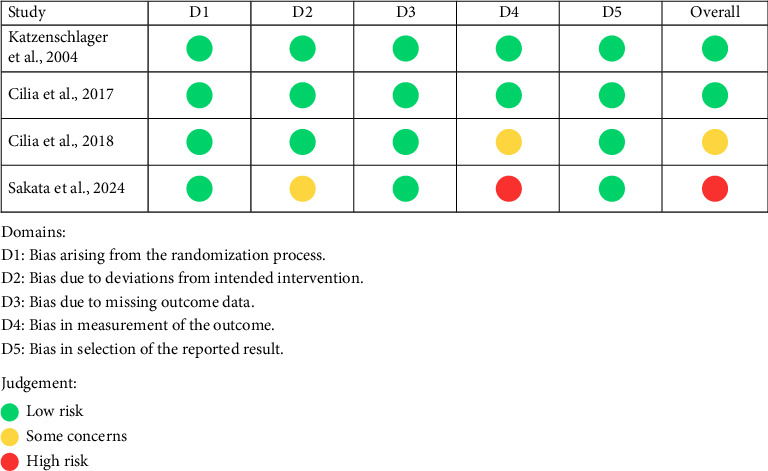
Risk of bias assessment across domains for each randomized controlled trial (RCT) included in the systematic review, using the RoB 2 tool.

**Figure 3:**
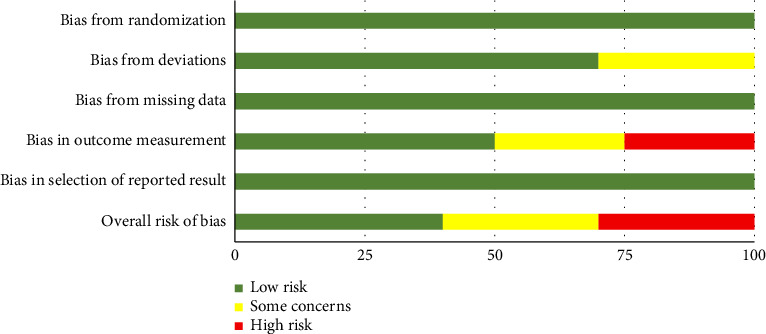
Weighted bar plots of the distribution of risk of bias judgments within each bias domain in overall studies.

The risk of bias for RCTs was assessed using the RoB 2 tool [28]. All RCTs were judged to have a low risk of bias regarding the randomization process, missing outcome data, and selection of reported results. However, the trial by Cilia et al. showed some concerns in the domain of outcome measurement, while Sakata et al. was rated as high risk in this domain. Regarding deviations from intended interventions, Sakata et al. was assessed as having some concerns, whereas the remaining trials had low risk of bias in this domain. Consequently, the studies by Katzenschlager et al. and Cilia et al. were rated as having an overall low risk of bias, Cilia et al. had some concerns, and Sakata et al. was judged to have a high overall risk of bias [31–34]. Domain-level risk of bias assessments for each RCT are summarized in Figure 2, and the overall distribution across domains is presented in Figure 3.

**Figure 4:**
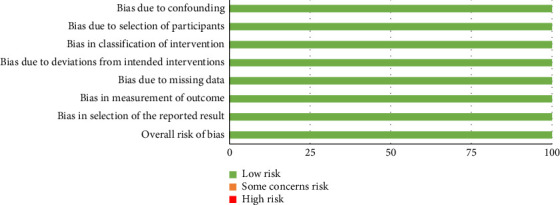
Risk of bias assessment across domains for the nonrandomized clinical trial included in the systematic review, using the ROBINS-I tool.

The risk of bias for the nonrandomized clinical trial was assessed using the ROBINS-I tool. This study was evaluated to have an overall low risk of bias [30]. The corresponding visualization is shown in Figure 4.

### 3.4. Efficacy Measure of *M. pruriens*

The efficacy measures were assessed using multiple outcomes, including clinical assessment, improvement in motor and nonmotor responses, and QoL in PD patients.

Table 3 showed the results for efficacy measures of *M. pruriens.*

### 3.5. Motor Responses

All included trials assessed changes in both voluntary and involuntary motor responses following *M. pruriens* treatment. In the 1995 multicenter trial titled “An Alternative Medicine Treatment for Parkinson's Disease: Results of a Multicenter Clinical Trial,” the UPDRS-III scores improved significantly, decreasing from 18.2 to 9.8 overall. This improvement was observed after *M. pruriens* administration in both LD-naïve patients and those previously treated with LD/CD [30]. Similarly, Katzenschlager et al. reported the highest UPDRS scores during the “on” phase after administering 30 g of *M. pruriens*, with statistically significant improvements in the onset (*p*=0.046) and duration of the “on” state compared to LD/CD (*p*=0.021) [31]. Cilia et al. found that low and high doses of *M. pruriens*, particularly when combined with a DDCI (*M. pruriens* + DDCI), led to motor performance improvements—16% with *M. pruriens*-Ld and up to 50% with *M. pruriens* + DDCI [32]. In contrast, Cilia et al. reported no significant difference in MDS–UPDRS scores between *M. pruriens* and LD in the ITT population, which includes all participants originally allocated to the treatment groups, regardless of whether they completed the study or fully adhered to the protocol, though the PP group, including only those participants who completed the study according to the protocol without major deviations, did show motor improvements with *M. pruriens* [33]. Sakata et al. also observed no significant differences in UPDRS-III scores between *M. pruriens* and LD/CD, though *M. pruriens* treatment was associated with a faster onset (40 min vs. 53.6 min) and longer duration of the “on” state (356.4 min vs. 162.1 min) [34].

Four studies assessed involuntary movements using the AIMS scale. Katzenschlager et al. found no significant differences in AIMS scores between treatment arms [31]. However, Cilia et al. demonstrated that *M. pruriens* significantly reduced dyskinesia at both 90 and 180 min compared to LD + DDCI (e.g., *p*=0.021 for *M. pruriens*-Hd vs. LD + DDCI at 90 min) [32]. Cilia et al. reported a reduction in “on” time with troublesome dyskinesia in the *M. pruriens* group, though this did not reach statistical significance in the ITT or PP analyses [33]. Similarly, Sakata et al. found no significant difference in modified AIMS scores between the two treatments (*p*=0.8) [34].

### 3.6. Nonmotor Response

Only one study evaluated nonmotor symptoms following *M. pruriens* treatment using the NMSQ and the Movement Disorder Society–Unified Parkinson's Disease Rating Scale Part I (MDS–UPDRS-I) [33]. Although the results did not reach statistical significance, *M. pruriens* was found to be noninferior to LD/CD in both the ITT and PP populations. Specifically, the NMSQ total score was 8.7 in the ITT group (*p*=0.908) and improved to five in the PP group (*p*=0.188). Likewise, MDS–UPDRS-I scores were lower in the PP population, with a trend toward better performance in activities related to daily living and QoL (*p*=0.059).

### 3.7. QoL

Three trials assessed QoL-related outcomes following *M. pruriens* treatment [30, 32, 33]. The 1995 multicenter trial by the HP-200 in Parkinson's Disease Study Group evaluated UPDRS Parts I and II, which measure mentation/behavior and activities of daily living, respectively [30]. This study showed notable improvements after *M. pruriens* treatment after *M. pruriens* administration in both LD-naïve patients and those previously treated with LD/CD. Specifically, the UPDRS-I score decreased overall, indicating better cognitive and behavioral function, with more pronounced improvements in LD-naïve patients. Similarly, UPDRS-II scores, reflecting motor aspects of daily living, were substantially reduced, suggesting enhanced patient independence. In contrast, Cilia et al. reported a relatively higher UPDRS-II score during the off state, which may reflect differences in patient populations or treatment protocols [32]. Meanwhile, Cilia et al. found no statistically significant difference in PDQ-39 scores—a PD-specific QoL questionnaire—between LD/CD and *M. pruriens* treatments, although trends favored LD/CD in the ITT group and *M. pruriens* in the PP group [33]. These findings suggest that *M. pruriens* may offer comparable benefits to conventional therapy in terms of QoL, yet larger and more rigorously designed studies are necessary to confirm these effects.

### 3.8. Therapy Complication

Therapy-related complications were assessed using the UPDRS-IV scale in the study by Cilia et al., which showed a decrease from 4.2 during the off phase to 1.4 during the on phase [32].

### 3.9. Disability

Three studies assessed disability outcomes using the H&Y scale [30, 32, 33]. In the 1995 multicenter clinical trial “An alternative medicine treatment for Parkinson's disease: results of a multicenter clinical trial. HP-200 in Parkinson's Disease Study Group,” the H&Y scale decreased from 2.5 to 1.6 for the overall patient group, including both LD-naïve patients and those who discontinued LD/CD prior to the trial [30]. Similarly, Cilia et al. reported a mild to moderate stage of disability during the off phase, with a mean H&Y score of 2.6 [32]. In contrast, Cilia et al. found a comparable H&Y score of 2 during the on phase for both treatment groups, indicating bilateral or midline involvement without balance impairment [33].

### 3.10. LD Pharmacokinetics

Two trials evaluated pharmacokinetic parameters, including the area under the curve (AUC), peak LD concentration (*C*_max_), time to peak concentration (*T*_max_), and elimination half-life (*T*_*k*_) [31, 34]. Katzenschlager et al. reported significant differences when comparing LD with 30 g of *M. pruriens*. Specifically, *M. pruriens* treatment resulted in a higher AUC (43,087 ng.h/mL for *M. pruriens* vs. 16,243 ng·h/mL for LD), an increased *C*_max_ (14,606 ng/mL for *M. pruriens* vs. 6956 ng/mL for LD), and a shorter *T*_max_ (72.4 min for *M. pruriens* vs. 95.5 min for LD), while the elimination half-life (*T*_*k*_) remained similar at approximately 90 min [31]. Likewise, Sakata et al. observed significant increases in AUC (11,186 for *M. pruriens* vs. 4744 ng·h/mL for LD) and *C*_max_ (7607 for *M. pruriens* vs. 3095 ng/mL for LD) with *M. pruriens* compared to LD, although no significant difference was found for *T*_max_ (25.7 min for *M. pruriens* vs. 34.4 min for LD) [34]. Please refer to Table 4 for LD pharmacokinetics results.

### 3.11. Results of Safety Measure

The assessment of safety outcomes varied across the included studies. Three studies, “An Alternative Medicine Treatment for Parkinson's Disease: Results of a Multicenter Clinical Trial. HP-200 in Parkinson's Disease Study Group” conducted in 1995 [30], the Katzenschlager et al. trial in 2004 [31] and Cilia et al. in 2018 [33], primarily reported the occurrence of AEs, while one study conducted by Cilia et al. in 2017 compared AEs between multiple treatments [32]. The 1995 multicenter clinical trial (“An alternative medicine treatment for Parkinson's disease: results of a multicenter clinical trial. HP-200 in Parkinson's Disease Study Group”) did not observe any dyskinesia in either the LD-naïve group or the LD/CD-treated groups receiving *M. pruriens*; however, nausea, vomiting, and insomnia were noted in both groups [30]. Katzenschlager et al. reported that one patient withdrew due to brief vomiting after consuming 30 g of *M. pruriens*. Among the patients who completed the trial, two experienced nausea following 30 g *M*. *pruriens* treatment, and one reported dizziness after taking 15 g *M. pruriens.* Additionally, *M. pruriens* treatment showed a faster onset and longer duration without an increase in AEs [31]. The study by Cilia et al. conducted in 2018 further reported symptoms such as revulsion, nausea, excessive daytime somnolence, dizziness, worsening of PD symptoms, psychiatric effects, and other prolonged events in patients continuing *M. pruriens* treatment; some patients discontinued the trial due to dizziness or other adverse effects [33]. However, in the 2017 study by Cilia et al., AEs were compared across multiple treatments. There were fewer AEs associated with *M. pruriens* treatment, particularly reduced dyskinesia (*p*=0.021) and fewer gastrointestinal complaints; however, there was excessive daytime somnolence occurring significantly more often in the *M. pruriens* group compared to LD + DDCIs [32]. Safety findings are shown in Table 1.

## 4. Discussion

This systematic review synthesized evidence from clinical trials investigating the efficacy and safety of *M. pruriens* in PD. The findings generally suggest *M. pruriens*, which contains L-dopa along with other bioactive compounds, may help manage PD symptoms and treatment-related complications. *M. pruriens* treatment was associated with a faster onset of therapeutic effect, a longer duration of the “on” state, and a lower incidence of AEs—with no cases of dyskinesia reported in the included studies.

There was a significant potential in disease symptom improvement following *M. pruriens* treatment. The primary mechanism behind *M. pruriens* is the capacity of restoring dopamine deficiency in the striatum, characteristic of motor symptoms, due to the presence of L-dopa [35]. Moreover, *M. pruriens* constituents exhibit anti-inflammatory and antioxidative properties, can potentially slow the neuronal damage progression, and reduce neurotransmitter deficiency, which may help in alleviating nonmotor and QoL symptoms such as cognitive impairment and depression [36, 37].

Results showed an improvement in motor response among PD patients. Rossi et al. studied the physiopathology of PD motor responses [38]. They found that these symptoms are related to dopamine depletion in the basal ganglia, specifically in the putamen, caudate, globus pallidus, subthalamic nucleus, and nucleus accumbens [39]. *M. pruriens* extract showed the presence of L-dopa. It can cross the BBB, bind to dopamine receptors at the resident dopaminergic neurons in the striatum, and restore the neurotransmission [35].

It is important to emphasize that the clinical studies included in this review did not directly investigate the underlying mechanisms by which *M. pruriens* may exert neuroprotective or symptomatic effects. The mechanistic explanations often cited in the literature are based on preclinical evidence from animal and cellular models rather than from human trials. These mechanisms include antioxidant, anti-inflammatory, and antiapoptotic properties that could contribute to the neuroprotective potential of *M. pruriens* [17, 24]. For instance, studies in rodent models of PD have demonstrated that *M. pruriens* extracts can reduce oxidative stress markers, preserve mitochondrial function, and mitigate dopaminergic neuron loss [25]. Similarly, in vitro experiments have highlighted *M. pruriens'*s capacity to modulate pathways involved in neuroinflammation and apoptosis [24]. These preclinical findings provide a plausible biological rationale for the observed clinical improvements but remain speculative until confirmed in human molecular or cellular studies.

Following *M. pruriens* treatment, nonmotor and QoL-related symptoms were effectively improved. Depression and memory loss impacted negatively the QoL; nevertheless, the primary nonmotor symptoms observed were gastrointestinal distress, sleep issues, and olfactory deficits [40]. The physiopathology here was caused by the degeneration of the serotonergic pathway and accumulation of *α*-synuclein in the olfactory bulb, gastrointestinal tract, and sleep-related structures in the brainstem [36, 40, 41]. This accumulation triggers the neuroinflammation and oxidative stress [42].

Usually, the inflammation arises from the stimulation of microglia by signaling molecules like nitric oxide “NO” and dysregulation of the NF-kB pathway. The activation of nitric acid synthase “NOS” stimulates the production of prostaglandin E2 “PGE2,” an inflammatory mediator [43]. Additionally, the activation of the NF-kB pathway stimulates the expression of oxides such as ROS and proinflammatory cytokines (such as TNF-*α*), causing neuronal death via oxidative stress as a secondary neurotoxicity [43–45].

Studies have shown that *M. pruriens* extract contained polyphenols and ursolic acid and glutathione protecting against the membrane peroxidation, ethanol, NADH, and CoQ10 [20, 46]. They can reduce the production of NO and NOS by inhibiting iNOS expression in the substantia nigra and the striatum and improve tyrosine hydroxylase responsible for L-dopa generation in the basal ganglia [24]. Additionally, UA regulates the NF-kB pathway by inhibiting its nuclear translocation and the expression of inflammatory cytokines (TNF-*α*), protecting neurons from inflammation, oxidative stress, and death in the basal ganglia [47]. This confers the anti-inflammatory and antioxidative properties of *M. pruriens* extract, potentially treating nonmotor and QoL symptoms related to PD [43].

Therefore, PD showed a high rate of apoptosis. Normally, the PI3K/AKT pathway and NO regulate apoptosis by balancing proapoptotic and antiapoptotic factors such as Bax and Bcl2, respectively [48]. Recent studies had shown the presence of ethanolic compounds in *M. pruriens* extract. Ethanol stimulates Bcl2 and pAkt1 expression along with Bax inhibition, regulating apoptosis [24].

*M. pruriens* extract also contains serotonin [47]. It can restore its concentration by protecting serotonergic neurons from death after their impairment in PD condition [20].

Gonzalez-Maldonado and his colleagues demonstrated an improvement in the nonmotor response in PD patients taking *M. pruriens* with a cup of green tea daily [49]. Another study that utilized *M. pruriens* cotyledon powder showed that NADH in *M. pruriens* can probably enhance mitochondrial complex I activity and the synthesis of dopamine [20]. These studies suggest a beneficial role of *M. pruriens*.

Disability is defined as any lack of capacity to perform a normal activity due to an impairment. Therapy complications and disability associated with disease progression and long-term use of treatment are assessed here, showing a reduction following *M. pruriens* treatment.

Dyskinesia is a common effect of prolonged PD treatment. Neta and his colleagues showed the role of serotonin in the long-term complications related to L-dopa treatment [25]. Furthermore, Sathiyanarayanan and Arulmozhi reported the antidyskinetic effects of serotonin present in *M. pruriens* extract [50]. Clinical assessment of patients with PD following *M. pruriens* treatment revealed a higher AUC with increased LD *C*_max_ with a shorter *T*_max_ compared to LD treatment. However, half-life elimination remained similar. Additionally, there was a longer duration of the on phase and a shorter time needed to reach it. It was explained by the accessibility of L-dopa and other chemicals and the capacity to easily reach the brain and restore neurotransmission.

The safety outcome indicated a reduction in the AEs among PD patients without any observed dyskinesia in the short-term treatment; however, the long-term and the tolerability of *M. pruriens* by all PD patients require further evaluation. Additionally, genistein found in *M. pruriens*'s seeds has been proposed as DDCI activity that improves the therapeutic potential of *M. pruriens* in PD [51].

### 4.1. Limitations and Future Directions

Our review had several limitations. Small sample sizes reduce the statistical power and generalizability of the results. Participants' diverse characteristics, including variations in age, disease severity, and symptom profiles, could impact the outcomes. Additionally, the heterogeneity in outcome measures across trials posed significant challenges for synthesizing results and performing meta-analysis. Short trial durations limit our understanding of the long-term effects and safety of *M. pruriens*. Potential publication bias and variations in methodological quality complicate findings interpretation. Therefore, larger, well-designed, and longer-term studies at the molecular and cellular level are needed to confirm the efficacy and safety of *M. pruriens* in humans. It is advised to promote collaborations to share findings from *M. pruriens* studies, to standardize *M. pruriens* formulation and dose to ensure consistency in CT, and to put procedures in place for tracking unfavorable outcomes.

## 5. Conclusion

This systematic review of clinical trials suggests that *M. pruriens* may offer potential benefits in managing PD symptoms, including improvements in motor function and therapy-related complications. Further well-designed clinical studies are needed to confirm these findings and to evaluate the long-term efficacy and safety of *M. pruriens* in PD management.

## Figures and Tables

**Table 1:** Study characteristics.

Author & year	Study type	Sample size (*N*)	Age (mean [SD])	Gender		PD duration (mean years)	Treatments	Dose	Outcomes	Efficacy findings	Safety findings
				Male (*N*)	Female (*N*)						
An Alternative Medicine Treatment for Parkinson's Disease: Results of a Multicenter Clinical Trial. HP-200 in Parkinson's disease study group, 1995 [30]	Clinical trial	60	59 (9)	46	14	4.1	HP-200 containing 33.33 mg levodopa per gram	- LD naïve group patients averaged 5 ± 2 sachets/day - Carbidopa/levodopa-treated patients averaged 7 ± 3 sachets/day 1 sachet = 1.5 g *M. pruriens*	Efficacy Safety	Improvement in voluntary motor response, quality-of-life-related symptoms, disability symptoms. Any observed dyskinesia.	There was no observed dyskinesia in either levodopa (LD)-naïve patients or those previously treated with LD/carbidopa (LD/CD) after *M. pruriens* administration; however, nausea, vomiting, and insomnia were noted in both groups

Katzenschlager et al. 2004 [31]	Randomized clinical trial	9	62.2	4	5	12.4	Levodopa/carbidopa, 15 or 30 g *M. pruriens*	15 g *M. pruriens* (≈500 mg levodopa) 30 g *M. pruriens* (≈1000 mg levodopa) Compared with 200 mg levodopa/50 mg carbidopa (standard LD/CD)	Efficacy Pharmacokinetics	Improvement in voluntary motor response but similar for involuntary motor response, “full on stage” with prolonged duration and reduced time to reach, increase in AUC & *C*_max_, reduced *T*_max_ but similar *T*_*k*_, some AEs observed	One patient withdrew due to brief vomiting after consuming 30 g of *M. pruriens*. Among the patients who completed the trial, two experienced nausea following 30 g of *M. pruriens* treatment, and one reported dizziness after taking 15 g of *M. pruriens*
Cilia et al. 2017 [32]	Randomized clinical trial	18	61.8 (9.10)	13	5	9.8	Levodopa, *M. pruriens*, dopamine agonists, iMAO-B, iCOMT, amantadine, anticholinergics	Low-dose (*M. pruriens*-Ld): 12.5 mg/kg of levodopa from *M. pruriens* High-dose (*M. pruriens*-Hd): 17.5 mg/kg of levodopa from *M. pruriens*	Efficacy Safety	Improvement in voluntary motor response, involuntary motor response, and quality-of-life-related symptoms; reduction in therapy-related complications; no improved disability; AEs observed	Adverse events were compared across multiple treatments, with excessive daytime somnolence occurring significantly more often in the *M. pruriens* group compared to LD plus dopa-decarboxylase inhibitors (LD + DDCIs)

Cilia et al. 2018 [33]	Randomized clinical trial	14	61.1(10)	11	3	9.4	LD, *M. pruriens*	The mean levodopa dose from *M. pruriens* was 4144.7 mg/day, consistent with the 5.7% calculation	Efficacy Safety	Improvement of voluntary motor response in ITT and PP populations, improvement in quality-of-life-related symptoms, improvement in nonmotor response in PP population but not in ITT, similar score for disability scale, reduced time of on phase with troublesome, some AEs observed	There were reported symptoms such as revulsion, nausea, excessive daytime somnolence, dizziness, worsening of PD symptoms, psychiatric effects, and other prolonged events in patients continuing *M. pruriens* treatment; some patients discontinued the trial due to dizziness or other adverse effects

Sakata et al. 2024 [34]	Randomized clinical trial	7	66.1 (7.2)	2	5	11.2	LD/CD, *M. pruriens*	Each patient received a single dose of 100/10 mg LD/CD tablets, and 11 g of *M. pruriens* reagent contained 442.2 mg of levodopa	Efficacy	Improvement in voluntary motor response but similar score for involuntary motor response, “full on stage” with prolonged duration and reduced time to reach increase in AUC and C max, reduced *T*_max_	

*Note: N* = number, LD = levodopa, LD/CD = levodopa/carbidopa, *M. pruriens* = *Mucuna pruriens*, HP-200 = an extract from *Mucuna pruriens* containing L-dopa, AUC = area under the plasma concentration curve, *C*_max_ = the peak plasma levodopa concentration, *T*_max_ = time to peak, *T*_*k*_ = apparent elimination half-time, ITT = intention-to-treat population, PP = per-protocol population, IMAO-B: monoamine oxidase-B inhibitors, iCOMT = catechol-O-methyltransferase inhibitor, LD + DDCI = levodopa with dopa-decarboxylase inhibitor.

Abbreviations: AEs = adverse events, PD = Parkinson's disease, SD = standard deviation.

**Table 2:** Summary of the studies about *M. pruriens* effects in PD patients.

Article	Parkinson's disease symptoms					
	Motor symptoms		Nonmotor symptoms	Quality of life	Disability	Therapy complications
	Voluntary	Involuntary				
(“An Alternative Medicine Treatment for Parkinson's Disease: Results of a Multicenter Clinical Trial. HP-200 in Parkinson's Disease Study Group,”) [30]	✔			✔	✔	
Katzenschlager et al. [31]	✔	✔				
Cilia et al. [32]	✔	✔		✔	✔	✔
Cilia et al. [33]	✔	✔	✔	✔	✔	
Sakata et al. [34]	✔	✔				

**Table 3:** Results of efficacy outcome.

PD symptoms	Article	Scale	Findings
Voluntary motor symptoms	An Alternative Medicine Treatment for Parkinson's Disease: Results of a Multicenter Clinical Trial. HP-200 in Parkinson's Disease Study Group [30]	UPDRS-III	UPDRS-III scores decreasing after *M. pruriens* administration from 18.2 to 9.8 overall demonstrated an improvement in both levodopa (LD)-naïve patients and those previously treated with levodopa/carbidopa (LD/CD)
	Katzenschlager et al. [31]	UPDRS	The highest UPDRS scores were during the “on” phase after administering 30 g of *M. pruriens*, with statistically significant improvements in the onset (*p*=0.046) and duration of the “on” state compared to LD/CD (*p*=0.021)
	Cilia et al. [32]	UPDRS-III	Low and high doses of *M. pruriens*, particularly when combined with a dopa-decarboxylase inhibitor (*M. pruriens* + DDCI), led to motor performance improvements—16% with *M. pruriens*-Ld and up to 50% with *M. pruriens* + DDCI
	Cilia et al. [33]	MDS–UPDRS	There is no significant difference in MDS–UPDRS scores between *M. pruriens* and LD in the intention-to-treat (ITT) population, though the per-protocol (PP) group did show motor improvements with *M. pruriens*
	Sakata et al. [34]	UPDRS-III	There are no significant differences in UPDRS-III scores between *M. pruriens* and LD/CD, though *M. pruriens* treatment was associated with a faster onset (40 min vs. 53.6 min) and longer duration of the “on” state (356.4 min vs. 162.1 min)

Involuntary motor symptoms	Katzenschlager et al. [31]	AIMS	There are no significant differences in AIMS scores between treatment arms (LD, 15 g *M. pruriens,* and 30 g *M. pruriens*)
	Cilia et al. [32]	Dyskinesia measure	*M. pruriens* significantly reduced dyskinesia at both 90 and 180 min compared to LD + DDCI (e.g., *p*=0.021 for *M. pruriens* -Hd vs. LD + DDCI at 90 min)
	Cilia et al. [33]	Dyskinesia measure	There is a reduction in “on” time with troublesome dyskinesia in the *M. pruriens* group, though this did not reach statistical significance in the ITT or PP analyses
	Sakata et al. [34]	Modified AIMS	There is no significant difference in modified AIMS scores between the two treatments LD, and *M. pruriens* (*p*=0.8)

Non motor response	Cilia et al. [33]	NMSQ	The NMSQ total score was 8.7 in the ITT group (*p*=0.908) and improved to 5 in the PP group (*p*=0.188)
		MDS–UPDRS	MDS–UPDRS-I scores were lower in the PP population, with a trend toward better performance in activities related to daily living and quality of life (*p*=0.059)

Quality of life	An alternative medicine treatment for Parkinson's disease: results of a multicenter clinical trial. HP-200 in Parkinson's Disease Study Group [30]	UPDRS-I	UPDRS-I score decreased overall, indicating better cognitive and behavioral function, with more pronounced improvements in LD-naïve patients
		UPDRS-II	UPDRS-II scores, reflecting motor aspects of daily living, were substantially reduced, suggesting enhanced patient independence.
	Cilia et al. [32]	UPDRS-II	There is a relatively higher UPDRS-II score during the off state, which may reflect differences in patient populations or treatment protocols
	Cilia et al. [33]	In PDQ-39 scores	There is no statistically significant difference in PDQ-39 scores—a PD-specific QoL questionnaire—between LD/CD and *M. pruriens* treatments, although trends favored LD/CD in the ITT group and *M. pruriens* in the PP group

Therapy complication	Cilia et al. [32]	UPDRS-IV	There is a decrease from 4.2 during the off phase to 1.4 during the on phase

Disability	An alternative medicine treatment for Parkinson's disease: results of a multicenter clinical trial. HP-200 in Parkinson's Disease Study Group [30]	The Hoehn and Yahr (H&Y)	The H&Y scale decreased from 2.5 to 1.6 for the overall patient group, including both levodopa-naïve patients and those who discontinued LD/CD prior to the trial
	Cilia et al. [32]	The Hoehn and Yahr (H&Y)	There is a mild to moderate stage of disability during the off phase, with a mean H&Y score of 2.6
	Cilia et al. [33]	The Hoehn and Yahr (H&Y)	There is a comparable H&Y score of 2 during the on phase for both treatment groups, indicating bilateral or midline involvement without balance impairment

*Note:* LD = levodopa, LD/CD = levodopa/carbidopa. *M. pruriens* = *Mucuna pruriens*, DDCI = dopa decarboxylase inhibitor, ITT = intention to treat population, PP = per-protocol population, UPDRS = Unified Parkinson's Disease Rating Scale, H&Y = Hoehn and Yahr scale.

Abbreviations: AIMS = Abnormal Involuntary Movement Scale, MDS–UPDRS = Movement Disorder Society–Unified Parkinson's Disease Rating Scale, PDQ-39 = Parkinson's Disease Questionnaire-39, QoL = quality of life.

**Table 4:** Levodopa pharmacokinetic measures.

**Pharmacokinetics**	**Article**	**LD/CD means (SD)**	***M. pruriens* mean (SD)**			**p** **value**		
			**11 g**	**15 g**	**30 g**		**Difference between LD/CD vs 15 g *M. pruriens***	**Difference between LD/CD vs 30 g *M. pruriens***

AUC (ng·h/mL)	Katzenschlager et al. [31]	16,243 (2543)		16,306 (4024)	43,087 (9735)		NS	0.012
	Sakata et al. [34]	4744 (929)	11,186 (1528)			< 0.05		

*C*_max_ (ng/mL)	Katzenschlager et al. [31]	6956(1098)		8608 (1979)	14,606 (2662)		NS	0.025
	Sakata et al. [34]	3095 (700)	7607 (1546)			< 0.05		

*T*_max_ (min)	Katzenschlager et al. [31]	95.5 (10.5)		61.8 (12.9)	72.4 (15.1)		0.04	NS
	Sakata et al. [34]	34.3 (4.3)	25.7 (5.4)			0.3		

T1/2	Katzenschlager et al. [31]	90.8 (23.8)		58.6 (5.1)	94.0 (25.5)		NS	NS

*Note:* LD = levodopa, LD/CD = levodopa/carbidopa, *M. pruriens* = *Mucuna pruriens*, AUC = area under the plasma concentration curve, *C*_max_ = the peak plasma levodopa concentration, *T*_max_ = time to peak, *T*_*k*_ = apparent elimination half-time.

Abbreviation: SD = standard deviation.

## Data Availability

All data generated or analyzed during this study are included in this published article. As this is a systematic review, the data consist of published literature identified through database searches and are available from the corresponding author upon reasonable request.
